# Deficient arsenic methylation and global proteomic reprogramming in human keratinocytes during arsenic-induced skin carcinogenesis

**DOI:** 10.1007/s00204-026-04478-w

**Published:** 2026-06-27

**Authors:** Alexandra N. Nail, Mayukh Banerjee, Manting Xu, Caitlin H. Reynolds, Miroslav Stýblo, Peter H. Cable, Daniel W. Wilkey, Michael L. Merchant, Ana P. Ferragut Cardoso, Shelia D. Thomas, J. Christopher States

**Affiliations:** 1 Department of Pharmacology and Toxicology, University of Louisville, Louisville, KY USA; 2 Center for Integrative Environmental Health Sciences, University of Louisville, Louisville, KY USA; 3 Department of Nutrition, Gillings School of Global Public Health, University of North Carolina at Chapel Hil, Chapel Hill, NC USA; 4 Division of Nephrology and Hypertension, Department of Medicine, University of Louisville, Louisville, KY USA; 5 Department of Pharmacology and Toxicology, University of Louisville School of Medicine, 505 S. Hancock St. Rm 304, Louisville, KY 40202 USA

**Keywords:** Arsenic, Proteomics, Arsenic metabolism, Human keratinocytes, Arsenite methyltransferase

## Abstract

Chronic inorganic arsenic (iAs) exposure affects > 220 million people worldwide and skin cancer is a hallmark of long-term iAs exposure. Limited information exists regarding arsenic methylation by human keratinocytes and how methylation influences skin carcinogenesis. Inorganic arsenite (iAs^III^) and its methylated metabolites disrupt diverse zinc finger proteins, leading to differential toxicity patterns. We examined arsenic methylation capacity in non-malignant human keratinocytes and interrogated proteomic remodeling across three stages of iAs^III^ induced malignant transformation using the well-established preclinical HaCaT model. Arsenic methylation was assessed by hydride generation cryotrapping inductively coupled-mass spectrometry and global proteomic changes were analyzed by tandem-mass tagging liquid chromatography-tandem mass spectrometry. Primary, hTERT-immortalized and HaCaT human keratinocytes exhibited negligible arsenic methylation, with iAs^III^ comprising at least 98.5% of total intracellular arsenic, attributable to minimal expression of arsenite methyltransferase. Proteomic profiling identified over 275 differentially expressed proteins at each stage of transformation, including multiple zinc finger proteins implicated in cell cycle control, RNA metabolism, and genome stability. Ingenuity® Pathway Analysis revealed progressive, coordinated disruption of cancer-associated pathways and regulatory networks over the transformation timeline, including zinc-coordinating upstream regulators that may explain widespread pathway dysregulation. Collectively, our findings suggest that iAs^III^ promotes skin carcinogenesis by disrupting C3H1- and C4-type zinc finger protein-centered regulatory networks that coordinate cancer-associated signaling and metabolic pathways in human keratinocytes, highlighting key candidates for future mechanistic studies.

**Supplementary Information:**

The online version contains supplementary material available at https://doi.org/10.1007/s00204-026-04478-w.

## Introduction

Chronic exposure to inorganic arsenic (iAs; arsenite and arsenate) in drinking water affects roughly 220 million people worldwide, and non-melanoma skin cancer is a well-established consequence of long-term iAs exposure (Nail et al. 2022). Defining the molecular pathways that connect iAs exposure to skin carcinogenesis has been hindered by the absence of an animal model that develops skin cancer from standalone iAs exposure (Tokar et al. 2010). HaCaT cells chronically exposed to inorganic arsenite (iAs^III^) is an established preclinical model widely employed to study molecular mechanisms of iAs^III^-induced cutaneous squamous cell carcinoma (cSCCBanerjee et al. 2022a; Pi et al. 2008; Sun et al. 2009). Non-malignant HaCaT cells continuously exposed to iAs^III^ (100 nM sodium arsenite; NaAsO_2_) for 28 weeks fully transform and acquire an aggressive cSCC-like phenotype (Pi et al. 2008), demonstrate epithelial to mesenchymal transition (Banerjee et al. 2021), and form tumors when injected into immunocompromised mice (Alexander et al. 2019; Barajas-Olmos et al. 2019). Recently, high-throughput transcriptomics data revealed a detailed, molecular portrait across the timeline of HaCaT malignant transformation including differential mRNA expression (Banerjee et al. 2021, 2022a), differential alternative splicing (Ferragut Cardoso et al. 2022), and stochastic and deterministic gene expression changes (Banerjee et al. 2024b), driven by chronic iAs^III^ exposure. These studies determined that the gene expression landscape is not static but instead evolves longitudinally during iAs^III^-induced cSCC development (Banerjee et al. 2021, 2024a). This longitudinal approach is essential to develop a comprehensive understanding of the molecular events by which iAs^III^ initiates carcinogenesis and promotes malignant transformation of human keratinocytes. However, stepwise changes in the proteomic expression landscape during iAs^III^-induced malignant transformation remain unexplored, representing a critical knowledge gap.

Proteome-wide expression changes are observed in iAs^III^-exposed human keratinocyte lines and in arsenic-induced keratotic tissue (Guo et al. 2016; Mir et al. 2017; Udensi et al. 2014). Although foundational, these studies have major limitations that prevent successful extrapolation to explain how environmental iAs^III^ exposure initiates skin carcinogenesis. One study utilized a therapeutic iAs^III^ concentration (500 ppb arsenic trioxide; As_2_O_3_; equivalent to 2.5 µM iAs^III^; 8 passages) (Udensi et al. 2014) while all studies performed proteomics at a singular time point, limiting insight into sequential protein expression changes underlying cellular transformation. Comprehensive longitudinal proteomic profiling coupled with functional enrichment analysis is required to identify interconnected cellular pathways and networks dysregulated by chronic iAs^III^ exposure that drive iAs^III^-induced malignant transformation of human keratinocytes.

Arsenic-induced gene expression changes and toxic effects vary depending on the specific arsenic metabolite (Calatayud et al. 2013; Smeester et al. 2017; Su et al. 2006). In particular, iAs^III^ exposure alters the expression of a unique set of genes compared with methylated arsenic metabolites in each experimental setting (Smeester et al. 2017; Su et al. 2006). Furthermore, iAs^III^ and methylated arsenic metabolites differentially interact with target proteins, leading to distinct toxicological outcomes. For example, while iAs^III^ binds and displaces zinc from 3 cysteine and 1 histidine (C3H1) or 4 cysteine (C4) containing zinc finger proteins, monomethylarsonous acid (MMA^III^) can additionally target those with 2 cysteine and 2 histidine (C2H2) residues (Vergara-Geronimo et al. 2021; Zhou et al. 2014). Therefore, it is crucial to consider arsenic methylation status while trying to understand molecular mechanisms that promote iAs^III^-induced cSCC.

Pi et al. revealed that HaCaT cells do not methylate iAs^III^ (2.5 µM), suggesting that iAs^III^ itself drives malignant transformation in the HaCaT cell line (Pi et al. 2008). However, it is unknown if poor arsenic methylation is unique to HaCaT cells or is a core characteristic of human keratinocytes. Determining whether arsenic metabolism (or its absence) is a common feature across human keratinocytes will clarify: (i) Which arsenic metabolite(s) drive keratinocyte malignant transformation; (ii) which molecular targets driving arsenic-induced initiation and progression; and (iii) whether the HaCaT model is relevant for studying the molecular basis of arsenic-induced cSCC.

To address these questions, arsenic speciation analysis was performed across a panel of human keratinocytes representing primary (HEKn), hTERT-immortalized (Ker-CT) and spontaneously immortalized (HaCaT) cell lines. Expression of arsenite methyltransferase (AS3MT), the enzyme responsible for arsenic methylation, was also assessed across cell lines. Furthermore, the well-established preclinical HaCaT cell model of chronic iAs^III^-induced cSCC-like development was leveraged to determine longitudinal differential protein expression [7-week (pre-initiation changes), 19-week (initiation of transformation), and 28-week (fully transformed)] utilizing tandem-mass tagging liquid chromatography-tandem mass spectrometry (TMT-LC-MS/MS). Functional enrichment by pathway analysis was performed to provide biological context from individual protein level data by identifying dysregulated cellular processes, networks, and regulators as well as putative arsenic targets mediating the observed effects.

## Methods

### Chemicals

Sodium arsenite (iAs^III^ as NaAsO_2_; Chemical Abstracts Service Registry No. 7784 -0698; >99% pure) was obtained from Thermo Fisher Scientific Inc. (Waltham, MA, USA; RRID: SCR_008452; http://www.fishersci.com). Single-thaw aliquots of NaAsO_2_ were prepared monthly as 1000X stocks in ultrapure DNAse/RNase-free distilled water (Thermo Fisher Scientific Inc.; Cat #10977015), stored at − 20 °C and were thawed immediately before use. Trypsin-EDTA (0.05% trypsin-0.02% EDTA) used for passaging and penicillin/streptomycin were obtained from Thermo Fisher Scientific Inc (Cat # 25300054).

### Cell culture

HaCaT cells (RRID: CVCL_0038; https://web.expasy.org/cellosaurus/CVCL_0038) were a kind gift from Dr. Tai Hao Quan, University of Michigan. Ker-CT (CRL-4048; RRID: CVCL_S877; https://web.expasy.org/cellosaurus/CVCL_S877) and HepG2 hepatocellular carcinoma cells (HB-8065; RRID: CVCL_0027; https://web.expasy.org/cellosaurus/CVCL_0027) were obtained from the American Type Culture Collection (Manassas, VA). Neonatal human primary epidermal keratinocyte cell line HEKn (C0015C) from a single male donor were purchased from Thermo Fisher Scientific Inc. HaCaT, Ker-CT, HepG2, and HEKn cells were cultured as previously published (Bajsert et al. 2024; Ferragut Cardoso et al. 2022; Nail et al. 2022). For acute exposure experiments, 1 × 10^6^ cells were plated per 100-mm dish and exposed to iAs^III^ (NaAsO_2_; 100 nM; 24 h). Chronic iAs exposure (0/100 nM; 28 weeks) was performed in HaCaT cells (spontaneously immortalized human keratinocyte from a 62-year-old adult male)(Boukamp et al. 1988) as previously published by our group (Banerjee et al. 2021; Ferragut Cardoso et al. 2022). At the initiation of chronic exposure, HaCaT cells were randomly assigned to control or iAs^III^ exposed groups (0/100 nM iAs) in independent quadruplicates, providing power to empirically delineate effects of iAs exposure, passaging time and stochasticity (Banerjee et al. 2022a, 2024b). Chronic cultures were repeatedly tested for mycoplasma contamination along the malignant transformation timeline (MycoSensor PCR Assay Kit; Agilent Technologies; RRID: SCR_013575; http://www.agilent.com/; Cat # 302108) while the identity of HaCaT culture replicates was authenticated at the end of the chronic exposure by comparing short tandem repeat (STR) mapping results generated by Labcorp, (Burlington, NC) to the Cellosaurus database (≥ 80% match with reference STR profile)(Ferragut Cardoso et al. 2022).

### Analysis of arsenic species in cells and culture media

Cells were treated as described in the previous section. Media and cells were collected by scraping as per published protocols (Matousek et al. 2017). Samples (media and cells) were immediately shipped on dry ice overnight to the University of North Carolina at Chapel Hill for arsenic speciation analysis. Samples were analyzed by ICP-MS following published protocols standardized by the Styblo laboratory (Matousek et al. 2017). The ICP-MS operator was blinded to sample identity. Total arsenic concentration (tAs) in media and cells were calculated as the sum of iAs, mono- and di-methylated arsenic species (MMAs and DMAs) in media (pg/7 mL) and cells (pg/1 × 10^6^ cells collected in 0.5 mL in deionized water). Mass estimates for each species (iAs, MMAs and DMAs) were subsequently converted to nanomolar concentrations. Percentages of tAs represented by iAs (%iAs), MMAs (% MMAs), and DMAs (% DMAs) were calculated for media and cells to assess efficiency of arsenic methylation (Matousek et al. 2017).

### RNA isolation and cdna generation

Total RNA was purified using the miRVana RNA Isolation Kit (ThermoFisher Scientific Inc.; Cat # AM1561) as described previously (Banerjee et al. 2021). RNAs with 260/230 ratios outside of the normal range (2.0–2.2) indicating the presence of organic compounds, were precipitated using 2.5 M ammonium acetate and ethanol (Spivak and Hanawalt 1995) and redissolved in elution buffer solution provided within the miRVana RNA Isolation Kit. cDNA was generated from total RNA (100 ng) using the SuperScript IV First Strand Synthesis System (Thermo Fisher Scientific Inc.) as per the manufacturer’s instructions using Oligo(dT)_20_ primers.

### Reverse transcription-polymerase chain reaction (RT-PCR)

RT-PCR reactions were performed using primers designed specifically to amplify only the AS3MT canonical isoform (AS3MT-201; Supplementary Fig. 1A; Forward Primer: 5′ GACATGGCTGCACTTCGT 3′; Reverse Primer: 5′ CGTTGGTCTGGAGGTCTG 3′). For each sample, 200 ng of cDNA was amplified using 2 µM of each primer, Takara ExTaq® DNA Polymerase and ExTaq Buffer system (Cat # RR001A) in a 10 µL reaction volume. The cycling conditions were 95 °C for 1 min; followed by 36 cycles of: 95 °C for 30 s, 63 °C for 30 s, and 72 °C for 45 s; and, a final extension step at 72 °C for 5 min. Amplicons were resolved using 1.2% agarose gel electrophoresis, stained with RedSafe™ Nucleic Acid Staining Solution (Bulldog Bio; Portsmouth, NH.; Cat # 21141, 1x final concentration) followed by image acquisition (Ferragut Cardoso et al. 2022).

### Cloning and sequencing of AS3MT

RNA samples were treated with RNase-free DNase I (Thermo Fisher Scientific; Cat # EN0521), as per manufacturer’s instructions (1 unit/1000 ng DNA). Prior to RT-PCR reactions, 1250 ng RNA was first treated with DNase I or RNase-free DNase-free ddH_2_O (untreated control) and incubated at 37 °C for 30 minutes. Half of the reaction volume was then removed from each tube (625 ng RNA) and digested for a second time with DNase I or RNase-free ddH_2_O by incubation at 37°C for 30 minutes. To the other half of the reaction 50 mM EDTA was added (final concentration 5 mM EDTA), and the reaction was incubated at 65°C for 10 minutes to halt DNase I activity. Following the second DNase I reaction, untreated or DNase I-treated samples were subsequently treated again with 5 mM EDTA and incubated at 65°C for 10 minutes to halt DNase I activity. For RNase A reactions, RNA samples treated with or without DNase I were divided into separate tubes and digested with or without RNase A (4 µg/mL final concentration) by incubation at 37°C for 30 minutes. DNase I untreated or treated, and RNase A treated or untreated RNA samples were then stored at -80°C until further processing. cDNA was generated as described in the “RNA isolation and cDNA Generation” section. For each sample, 15 ng of cDNA was amplified using the same conditions described within the “RT-PCR” section. Amplicons were resolved on 6% polyacrylamide gels and subsequently isolated using the Nucleospin® Gel and PCR Clean-up System (Takara; Cat # 740609.250), ligated into pCR2.1 (The Original TA Cloning® Kit, Invitrogen; Cat # K202040), and transformed into TOP10F’ chemically competent *E. coli* (ThermoFisher Scientific; Cat # C303003). Blue/white colony screening was performed using X-Gal/IPTG and selection by ampicillin. Colonies were screened for inserts by colony PCR using M13 forward (5′GTTTTCCCAGTCACGAC 3′) and reverse (5’ CAGGAAACAGCTATGAC 3’) primers, and positive colonies were cultured for plasmid isolation (Nucleospin® Plasmid Prep, Takara; Cat # 740499.250) and subsequent Sanger sequencing (Eurofins Genomics, Louisville, KY). Sequences obtained were aligned to the *Homo sapiens* AS3MT gene (NM_020682.4) using NCBI BLAST (blastn; RRID: SCR_004870; http://blast.ncbi.nlm.nih.gov/Blast.cgi). The new AS3MT intron retaining isoform sequence was submitted to GenBank (RRID: SCR_002760; http://www.ncbi.nlm.nih.gov/Genbank/, Accession: PQ114255; URL: https://www.ncbi.nlm.nih.gov/nuccore/PQ114255).

### Proteomic analyses

Whole cell lysates were isolated from HaCaT cells chronically incubated with or without iAs^III^ exposure (0/100 nM) at three different time points (7, 19, 28 weeks). Briefly, cells were lysed in buffer containing 10 mM Tris-HCl (pH 7.4), 1 mM Na_2_EDTA, 0.1% sodium dodecyl sulfate (SDS), 1 mM phenylmethanesulfonyl fluoride (PMSF), 1 mM sodium vanadate, and 1X protease inhibitor cocktail (Roche cOmplete™ Protease Inhibitor Cocktail) prior to protein quantification and downstream proteomic analysis. Proteomic analysis was performed using the previously published TMT-LC-MS/MS workflow developed by our laboratory in conjunction with the University of Louisville Proteomics Technology Center (Ferragut Cardoso et al. 2022). Protein lysate samples were mixed with an equal volume of 10% wt/vol SDS and 100 mM triethylammonium biocarbonate (TEAB), pH 7.55. Protein digestion was performed using S-Trap Micro Spin columns (Protifi, LLC), which alleviated the need for sample concentration/desalting using C18 columns. Briefly, 75 µg protein was reduced with dithiothreitol (DTT; final concentration 20 mM) at 95 °C for 10 min, cooled to room temperature, and alkylated with iodoacetamide (IAA; final concentration 40 mM) prepared in LC-MS-grade water (ThermoFisher; Cat # W6-4) for 30 min in the dark at room temperature. MS-grade trypsin (Pierce) was added at a 1:25 enzyme-to-protein ratio (3 µg: 75 µg protein) and the samples were incubated according to the S-Trap protocol at 47 °C for 1 h.

Tryptic digests were resuspended in 75 µL LC-MS-grade water and peptide concentration was determined by absorbance at 205 nm using a Nanodrop 2000 (Thermo Fisher). Two readings were obtained per sample and averaged for a concentration determination using Nanodrop software Version 1.6 (Thermo Fisher). Aliquots containing 40 µg peptide were derivatized with TMT 10plex reagents (Thermo Fisher) and pooled prior to high-pH reversed-phase fractionation.

First-dimensional peptide separation was performed using a U3000SD ultra-high-performance liquid chromatography (uHPLC) system (ThermoFisher Scientific) equipped with an LPG-3400SD pump, WPS-3000SL analytical autosampler, TCC-3000SD column thermostat, VWD-3100 detector, and AFC-3000 fraction collector. Peptides were separated using a Waters VanGuard cartridge and BEH XBridge C18 column (5 µM, 3.0 × 150 mm) with Buffer A [5% vol/vol acetonitrile (CH_3_CN); 10 mM ammonium formate (NH_4_HCO_2_), pH 10] and Buffer B [90% vol/vol CH_3_CN; 10 mM NH_4_HCO_2_, pH 10] prepared using LC-MS-grade water and HPLC-grade NH_4_HCO_2_ (Fluka, Cat # 17843). The flow rate was maintained at 300 µL/min with a total run time of 95 min. Columns were equilibrated in 100% Buffer A prior to sample loading.

Concentrated and desalted peptide samples (100 µg) were dissolved in 5% CH_3_CN containing 10 mM NH_4_HCO_2_ (pH 10), loaded onto the Waters BEH column, and separated using the following gradient: 0% Buffer B from 0 to 1 min, 0 to 10% Buffer B over 4 min, 10% to 40% Buffer B over 60 min, 40% to 98% Buffer B over 10 min, 98% Buffer B for 5 min, 98% to 0% Buffer B over 0.1 min, and 0% Buffer B for 9.9 min. Elution was monitored at 214 nm. Fractions were collected into 2-mL Eppendorf tubes. Fraction 1 included the 5-min load volume. Fractions 2–19 were collected in 1.25 min intervals such that tube 2 contained fractions 2, 20, and 38 (each concatenated fraction equal the admixture of *i*, *i* + 18, and *i* + 36 fractions). Fraction 20 collected the last 6.25 min of eluate (1.875 mL of eluate). Sample tubes were transferred to a Thermo Electron speed-vacuum for drying. Dried peptide fractions were stored at − 80° C until LC-MS analysis.

Samples were resuspended in 2% CH_3_CN/0.1% formic acid, and LC-MS data were collected in a top-10 data-dependent analysis using the Orbitrap Elite-ETD system with a mass resolution of 60,000 (for the first-dimension mass spectrometry MS1 and MS2). Differential protein expression for the canonical isoforms was determined using ProteomeDiscoverer 2.2.0.388 with MASCOT Server Version 2.5.1 (Matrix Science) and SequestHT (ThermoFisher Scientific). The TMT-LC-MS/MS operator was blinded to exposure status of the samples. The proteomics data is available for download from the Mass spectrometry Interactive Virtual Environment data repository (MassIVE; RRID: SCR_013665; https://massive.ucsd.edu/ProteoSAFe/static/massive.jsp; Persistent Identifier: MSV000095626; URL: https://MSV000095626@massive-ftp.ucsd.edu/).

### Statistical analyses and data presentation

Statistical analyses for arsenic methylation across cell lines (Supplementary Fig. 1) were performed using GraphPad Prism 10 (GraphPad Software, San Diego, CA, USA; RRID: SCR_002798; http://www.graphpad.com/). Statistical analyses for protein expression data were performed across biological quadruplicates for each exposure condition and time point. Average raw protein abundance values and standard deviations for biological quadruplicates are provided in Supplementary Table 1. A protein was defined to be differentially expressed at *p* < 0.05 and no fold change threshold was applied (Supplementary Table 2). Ingenuity pathway analysis (IPA; Qiagen, Venlo, The Netherlands; RRID: SCR_008653; http://www.ingenuity.com/products/pathways_analysis.html) was performed to predict dysregulated canonical pathways and upstream regulators utilizing log_2_(Fold Change) expression values as input (Banerjee et al. 2021; Ekuban et al. 2025). Pathways and upstream regulators were considered to be activated at − log(*p* value) > 1.3 and z-score > 1, while they were considered to be inhibited at − log(*p* value) > 1.3 and z-score < − 1 (Banerjee et al. 2021; Ekuban et al. 2025). Graphs and heat maps were generated using GraphPad Prism 10. Venn diagrams were generated using Venn diagram plotter (https://omics.pnl.gov/software/venn-diagram-plotter).

### Data availability

All proteomic and sequencing data have been deposited to publicly available databases. Accession numbers are provided in sections described above. All other data/material generated as a part of this work are available upon request.

## Results

### Human keratinocytes have very limited arsenic methylation capacity

**Fig. 1:**
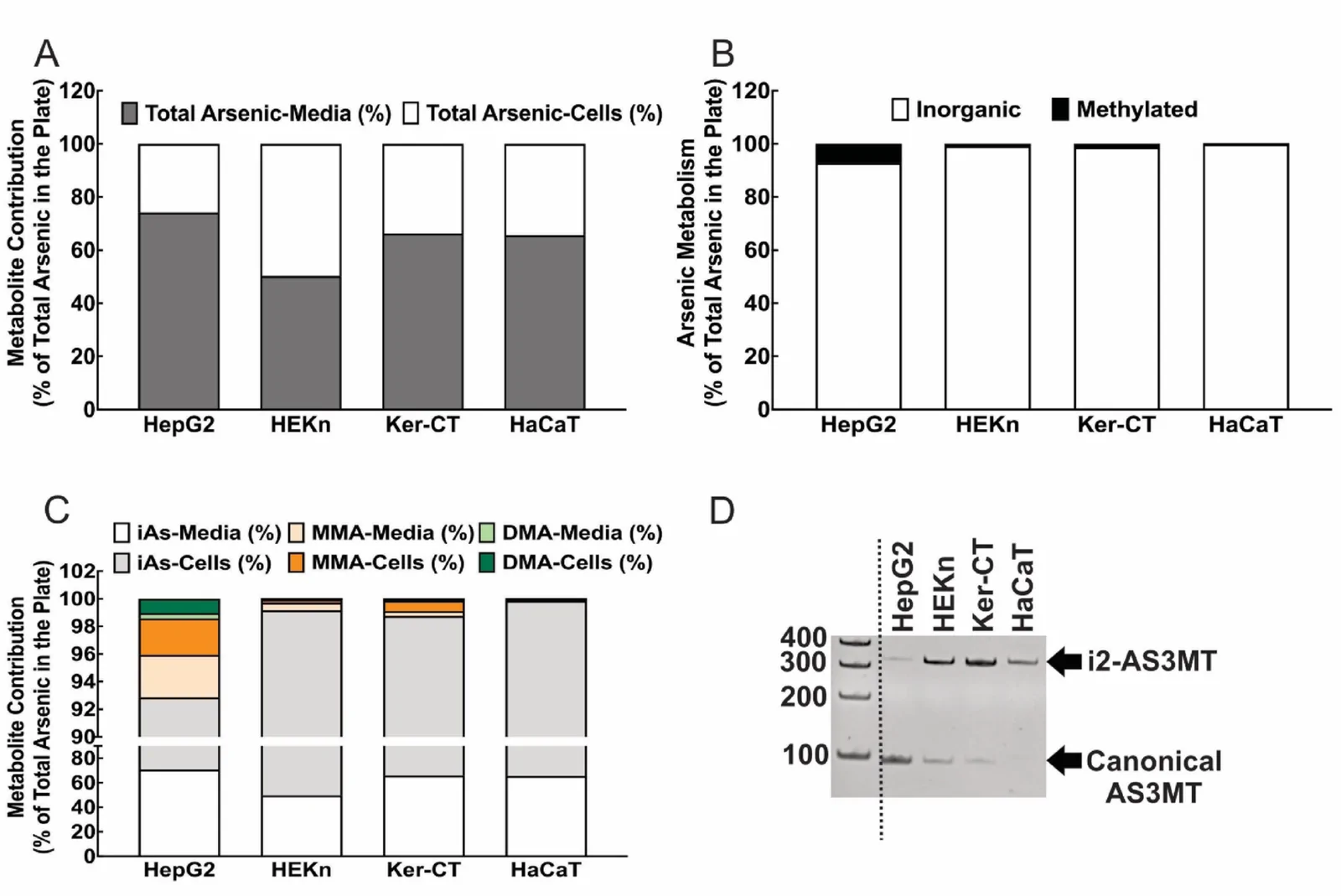
Human keratinocytes are deficient in arsenic methylation capacity. Human keratinocytes were exposed to iAs^III^ (100 nM) for 24 h and media and cell lysates were collected and analyzed by ICP-MS. **A** Percentage distribution of total arsenic inside the cells and in media across 3 human keratinocyte lines (HEKn, Ker-CT and HaCaT). **B** Percentage distribution of inorganic (iAs) and methylated (MMA and DMA) arsenic species across 3 human keratinocyte lines (HEKn, Ker-CT and HaCaT). **C** Percentage distribution of inorganic (iAs) and methylated (MMA and DMA) arsenic species inside cells and in the media demonstrating lack of arsenic methylation in human keratinocytes. **D** RT-PCR for canonical AS3MT isoform (AS3MT-201) across 3 human keratinocyte lines (HEKn, Ker-CT and HaCaT). A second band was observed at 332 bp in all samples, corresponding to a novel, intron 2 containing AS3MT mRNA isoform (i2-AS3MT). For panels** A**–**D**, established AS3MT expressing and arsenic methylating cell line HepG2 was used as positive control

To determine if human keratinocytes methylate arsenic, inorganic, mono- and di-methylated arsenic species were measured in media and in primary human keratinocytes (HEKn) and two immortalized, non-tumorigenic human keratinocyte lines (Ker-CT and HaCaT) exposed to iAs^III^ (100 nM; 24 h). HepG2 cells, which methylate arsenic, were used as positive control (Drobna et al. 2006). iAs and methylated arsenic metabolites were detected in both media and cells for each cell line (Fig. 1A). Each keratinocyte line showed minimal arsenic methylation compared to HepG2 cells (Fig. 1B & Supplementary Fig. 1). Methylated arsenic species were at the lower level of detectability in HaCaT cells and contributed ~ 1% of total arsenic species in HEKn and Ker-CT cells. By comparison, methylation efficient HepG2 cells had approximately 7% methylated arsenic species (Fig. 1B). In general, the combined contribution of methylated metabolites was minimal in all keratinocyte lines compared to HepG2 cells (Fig. 1C).

We investigated if the steady state expression of arsenite methyltransferase (AS3MT) could explain the observed minimal arsenic methylation capacity in keratinocytes (Fig. 1D). Each keratinocyte line expressed strikingly lower canonical AS3MT mRNA transcript (AS3MT-201; 94 bp fragment) compared to HepG2 cells and we were unable to detect any canonical AS3MT mRNA in HaCaT cells (Fig. 1D). Unexpectedly, we detected an additional unannotated amplification product (332 bp; Fig. 1D) despite intentionally designing our primers to exclusively amplify the annotated canonical isoform (Supplementary Fig. 2A). We performed sequential DNaseI and RNAseA digestions on total RNA samples followed by RT-PCR. The 332 bp amplicon persisted after DNase I but not RNase A digestion (data not shown), confirming it as a novel transcript rather than experimental artifact from residual genomic DNA contamination. Subsequent cloning and sequencing identified it as a novel intron 2 retaining isoform of AS3MT (Accession No: PQ114255). In silico translation using Expasy showed that this harbors a premature stop codon (Supplementary Fig. 2B) preventing translation to a functional AS3MT (Gasteiger et al. 2003; Kervestin and Jacobson 2012). Taken together, these results demonstrate that deficient arsenic methylation is a core feature shared by human keratinocytes.

### Chronic iAs^III^ exposure promotes differential protein expression patterns during iAs-induced malignant transformation

**Fig. 2:**
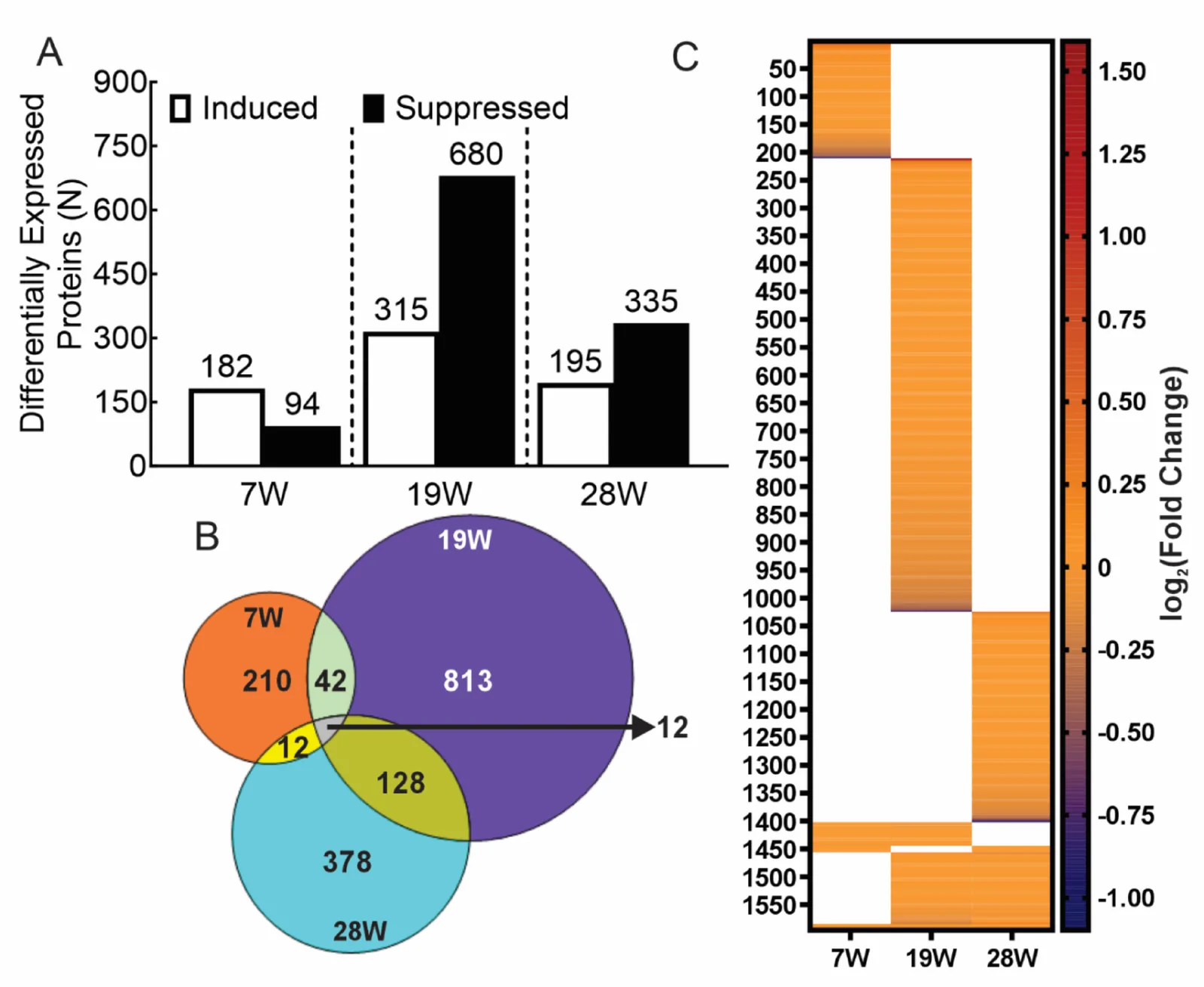
Chronic iAs^III^ exposure longitudinally alters global protein expression patterns in a pre-clinical HaCaT model for iAs-induced carcinogenesis. **A** Bar graph showing the number of differentially expressed proteins at 7-, 19-, and 28-week time points in HaCaT cells exposed to iAs^III^ (100 nM) compared with passage-matched unexposed controls (induced proteins, open bars; suppressed proteins, closed bars). **B** Venn diagram depicting the number of differentially expressed proteins at each time point (at 7-, 19-, 28-week) along the malignant transformation timeline along with overlaps. **C** Heat map of differentially expressed proteins at 7-, 19-, 28-week shown as log_2_ fold change expression. Numerals on the left y-axis refer to the serial numbers assigned to differentially expressed proteins in Supplementary Table 2. White areas signify that either a designated protein was not detected or differentially expressed at that time point. Differential protein expression was defined as p < 0.05; no fold change cut-off

iAs^III^ does not bind to nucleic acids but can bind to regulatory proteins altering their functions (Reaves et al. 2012). In addition to direct effects on protein function, chronic iAs^III^ exposure can induce widespread transcriptional and post-transcriptional remodeling during malignant transformation that may further influence protein abundance and regulatory signaling networks (Banerjee et al. 2021; Ferragut Cardoso et al. 2022). To define proteomic alterations associated with distinct stages of iAs^III^-induced transformation, we characterized longitudinal changes in the proteomic landscape utilizing the established preclinical HaCaT cell line model (Banerjee et al. 2021; Pi et al. 2008). Among 5393 total proteins detected in control and iAs^III^ exposed cells across the malignant transformation timeline, hundreds of proteins were differentially abundant at each time point (7-, 19-, 28-week time points) in iAs^III^-exposed cells compared to passage-matched, unexposed controls (Fig. 2A; Supplementary Table 2). The 19-week time point, which represents the initiation of transformation (Banerjee et al. 2021), contained the maximum number of differentially abundant proteins (Fig. 2A; Supplementary Table 2). Longitudinal comparison revealed that most differentially abundant proteins were unique at each time point, with limited overlap across time points (Fig. 2B–C; Supplementary Table 2). AS3MT protein was not detected at any time point with or without iAs^III^ exposure, consistent with RT-PCR results and previous transcriptomic data (Banerjee et al. 2021).

### Chronic iAs^III^ exposure dysregulates transformation-related pathways

**Fig. 3:**
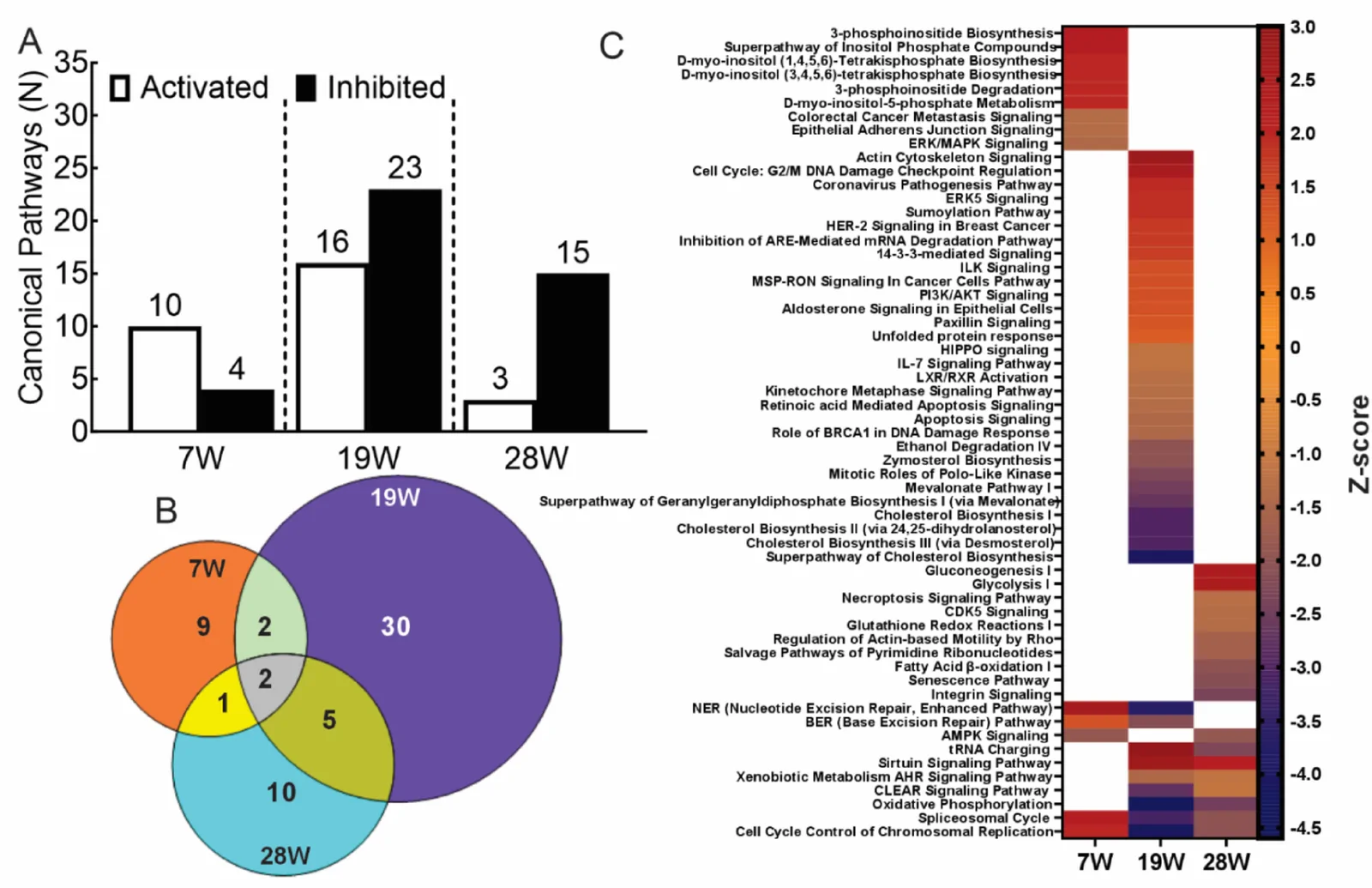
Chronic iAs^III^ exposure dysregulates multiple canonical pathways at each time point.** A** Bar graph showing the predicted number of activated/inhibited pathways at 7-, 19-, and 28-week time points in HaCaT cells exposed to iAs^III^ (100 nM) compared to passage-matched unexposed controls (activated pathways, open bars; inhibited pathways, closed bars). **B** Venn diagram depicting the number of predicted activated/inhibited pathways at each time point (at 7-, 19-, 28-week) along the malignant transformation timeline along with overlaps. **C** Heat map of predicted activated/inhibited pathways based on all differentially expressed proteins at 7-, 19-, and 28-week (presented in the same order as Supplementary Table 3). White areas signify that the canonical pathway was not predicted to be activated/inhibited at that time point. Activation of a pathway is defined as the − log(*p*-value) > 1.3; Z-score > 1, while inhibition is defined as − log(*p*-value) > 1.3; Z-score < − 1

To predict the canonical pathways dysregulated by chronic iAs exposure, IPA pathway analysis was performed using differentially abundant proteins as inputs. Multiple biochemical pathways were predicted to be activated/inhibited by chronic iAs^III^ exposure at each time point (Fig. 3; Supplementary Table 3). At 7-,19-, and 28-week time points, differentially abundant proteins populated 14 (10 activated, 4 inhibited), 39 (16 activated, 23 inhibited) and 18 (3 activated, 15 inhibited) dysregulated canonical pathways, respectively (Fig. 3A). Longitudinal comparisons indicated that iAs^III^ exposure dysregulated unique pathways at each time point with limited overlap (Fig. 3B–C). Only 2 canonical pathways were predicted to be dysregulated at all time points tested (Spliceosomal Cycle and Cell Cycle Control and Chromosomal Replication; Fig. 3B–C). These two canonical pathways were predicted to be activated at 7 weeks followed by inhibition at 19 and 28 weeks (Fig. 3C), highlighting that pathway functional status changes dynamically with time and stage of carcinogenesis.

### Chronic iAs^III^ exposure disrupts upstream regulator function

**Fig. 4:**
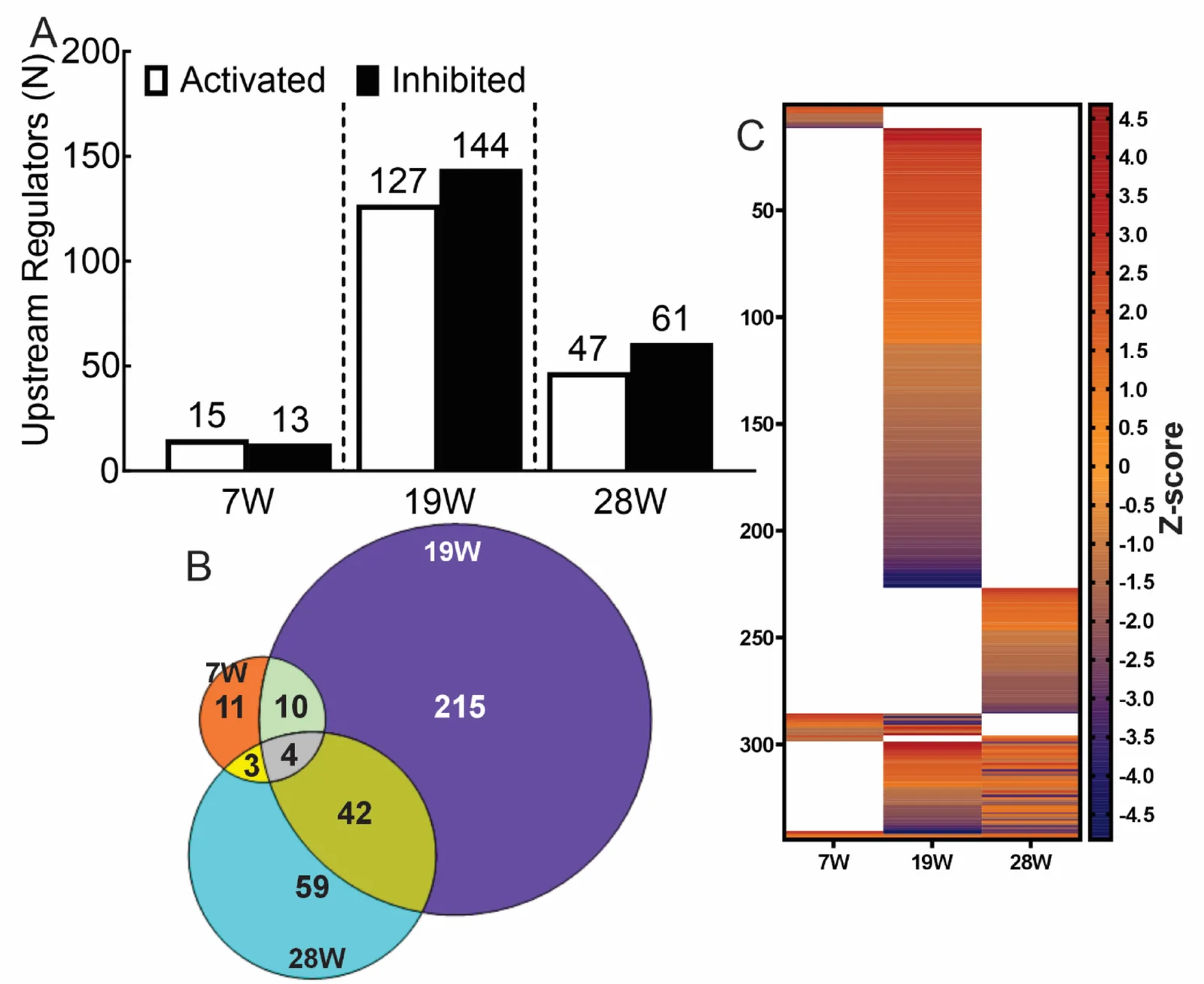
Chronic iAs^III^ exposure is predicted to dysregulate the function of multiple upstream regulators. **A** Bar graph showing the predicted number of upstream regulators with activated (open bars) or inhibited (closed bars) function at 7-, 19-, and 28-week time points in HaCaT cells exposed to iAs^III^ (100 nM) compared to passage-matched unexposed controls. **B** Venn diagram showing number of predicted activated/inhibited upstream regulators at each time point (at 7-, 19-, 28-week) along the malignant transformation timeline along with overlaps. **C** Heat map of predicted activated/inhibited upstream regulator function based on differential protein expression at 7-, 19-, and 28-weeks (presented in the same order as Supplementary Table 4). White areas signify that the upstream regulator was not predicted to be activated/inhibited at that time point. Upstream regulators were considered to be activated at − log(p value) > 1.3 and z-score > 1, while they were considered to be suppressed at − log(*p* value) > 1.3 and z-score< − 1

Next, IPA upstream regulator analysis was utilized to identify putative regulatory proteins whose activation/inhibition could explain the observed differential protein abundance in iAs^III^ exposed cells at each time point. Based on differentially abundant protein data, IPA predicted which upstream regulators were functionally activated/inhibited in accordance with known regulatory relationships in its database (Kramer et al. 2014). IPA predicted that 28 (15 activated, 13 inhibited), 271 (127 activated, 144 inhibited) and 108 (47 activated, 61 inhibited) upstream regulators were functionally dysregulated at 7-, 19- and 28- week time points, respectively (Fig. 4A; Supplementary Table 4). Most upstream regulators were predicted to be dysregulated at one time point only with relatively few overlaps (Fig. 4B–C), aligning with differentially expressed proteins and dysregulated canonical pathway data (Figs. 2 and 3). Only 4 upstream regulators [CCAAT Enhancer Binding Protein Beta (CEBPβ); COP9 Signalosome Subunit 5 (COPS5); KRAS-Protooncogene (KRAS); and E2F Transcriptional Factor 1 (E2F1)] were predicted to be dysregulated at all three time points (Fig. 4C; Supplementary Table 3). Established oncoproteins COPS5 and KRAS were predicted to be activated at all three time points (Fig. 4C, Supplemental Table 4).

### Differentially expressed proteins and dysregulated upstream regulators are enriched in zinc finger proteins

**Fig. 5:**
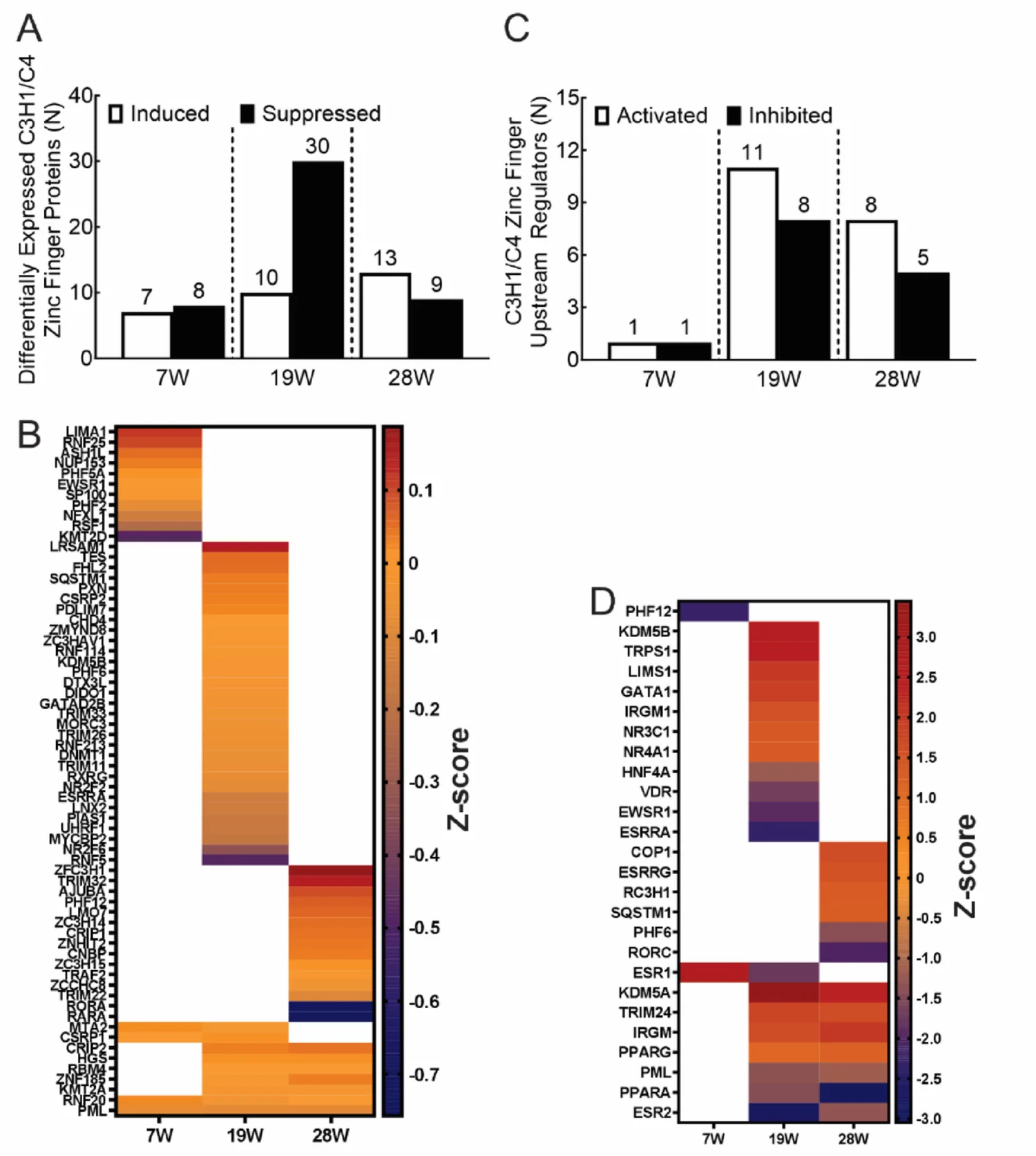
Multiple potential iAs^III^ binding target C3H1/C4 zinc finger proteins are differentially abundant and/or functionally dysregulated by chronic iAs^III^. **A** Bar graph showing the number of differentially abundant C3H1/C4 zinc finger proteins at 7-, 19-, and 28-week time points in HaCaT cells exposed to iAs^III^ (100 nM) compared with passage-matched unexposed controls (induced proteins, open bars; suppressed proteins, closed bars). **B** Heat map of differentially abundant C3H1/C4 zinc finger proteins at 7-, 19-, 28-weeks shown as log_2_ fold change abundance (presented in the same order as Supplementary Table 5). White areas signify that either a designated protein was not detected or differentially abundant at that time point. Differential protein expression was defined as *p* < 0.05; no fold change cut-off. **C** Bar graph showing the number of C3H1/C4 zinc finger upstream regulators with predicted activated (open bars) or inhibited (closed bars) function at 7-, 19-, and 28-week time points in HaCaT cells exposed to iAs^III^ (100 nM) compared with passage-matched unexposed controls. **D** Heat map of predicted activated/inhibited C3H1/C4 zinc finger upstream regulator function based on differential protein expression at 7-, 19-, and 28-weeks (presented in the same order as Supplementary Table 6). Upstream regulators were considered to be activated at − log(*p* value) > 1.3 and z-score > 1, while they were considered to be suppressed at − log(*p* value) > 1.3 and z-score< − 1

Trivalent arsenic can bind to zinc finger proteins and displace zinc (Zhou et al. 2014). Arsenic binding to target zinc finger proteins can alter their expression and/or function (Vergara-Geronimo et al. 2021). iAs^III^ specifically binds to zinc finger proteins that coordinate zinc ions between 3 cysteine and 1 histidine (C3H1) or four cysteine (C4) residues (Zhou et al. 2011). Given that arsenic in human keratinocytes existed almost exclusively in the inorganic trivalent form (Fig. 1), we sought to identify if C3H1/C4 zinc finger proteins were differentially abundant and if C3H1/C4 zinc finger containing upstream regulators were predicted to be functionally dysregulated (Fig. 5). To identify differentially abundant and/or functionally dysregulated C3H1/C4 zinc finger proteins, we intersected the list of differentially abundant proteins or dysregulated upstream regulators with a list of known human zinc finger proteins obtained from the Hugo Gene Nomenclature Committee database (HGNC) .

We found 66 C3H1/C4 zinc finger containing proteins were differentially abundant across all time points (Fig. 5A–B; Supplementary Table 5). At 7-, 19- and 28-week time points, 15 (7 induced; 8 suppressed), 40 (10 induced; 30 suppressed) and 22 (13 induced; 9 suppressed) C3H1/C4 zinc finger containing proteins were differentially abundant, respectively (Fig. 5A; Supplementary Table 5). RNF20 and PML were dysregulated at all time points tested (Fig. 5B; Supplementary Table 5). We also identified a total of 26 C3H1/C4 zinc finger containing upstream regulators that were predicted to be functionally activated/inhibited at least at one time point (Fig. 5C–D; Supplementary Table 6). At 7-, 19- and 28-week time points, IPA predicted 2 (1 activated; 1 inhibited), 19 (11 activated; 8 inhibited) and 13 (8 activated; 5 inhibited) C3H1/C4 zinc finger containing upstream regulators to be dysregulated, respectively (Fig. 5C; Supplementary Table 5). C3H1/C4 zinc finger proteins comprised 7.6% of all dysregulated upstream regulators predicted (26 out of 344). Unlike differentially abundant proteins, a large proportion (31%; 8 out of 26) of C3H1/C4 zinc finger upstream regulators were predicted to be dysregulated at two time points (Fig. 5D).

The intersection of zinc finger proteins in the human proteome with our datasets also identified a few C2H2 containing zinc finger proteins that were either differentially abundant or upstream regulators predicted to be dysregulated. We found 10 differentially abundant C2H2 zinc finger proteins (ZNF688, CTCF, WIZ, ZNF217, ZNF326, ZNF174, ZNF579, CHAMP1, VEZF1, ZNF277) and 6 C2H2 zinc finger regulators predicted to be functionally dysregulated (ZFP91, YY1, SNAI2, KLF5, SNAI1, ZNF106) across time points in our dataset. Thus, C3H1/C4 category proteins formed the overwhelming majority of differentially abundant zinc finger proteins and zinc finger containing upstream regulators predicted to be functionally dysregulated [66 out of 76 differentially expressed; (87%) and 26 out of 32 predicted to be functionally dysregulated; (81%)]. We also identified several zinc finger proteins that are established targets of iAs^III^-induced zinc loss and altered function both in the differentially abundant group [examples: PML Nuclear Body Scaffold (PML), Ring Finger Protein 20 (RNF20)], as well as the dysregulated upstream regulator group [PML Nuclear Body Scaffold (PML), GATA Binding Protein 1 (GATA1), and Sequestosome 1 (SQSTM1)] (Bercier et al. 2023; Medina et al. 2021; Shah et al. 2017; Zhang et al. 2014). Interestingly, six C3H1/C4 zinc finger proteins were both differentially expressed and predicted to be functionally dysregulated (EWSR1, SQSTM1, KDM5B, PHF6, ESRRA, PHF12, PML; Supplementary Tables 5 and 6).

Our previously published transcriptomic dataset (Banerjee et al. 2021) was reanalyzed for predicted upstream regulator function and integrated with predictions from the current proteomic dataset to identity overlapping patterns of dysregulation. Integration of transcriptomic and proteomic predictions revealed both shared and distinct patterns of predicted upstream regulator dysfunction across time points (Supplementary Tables 7 & 8). Shared predicted inhibition between transcriptomic and proteomic datasets included PHF12, TP53, NUPR1 at 7 weeks and FOXO3, VDR, and CEBPβ at 19 weeks, whereas E2F1 was the only predicted upstream regulator predicted to be activated (7 weeks). Although these upstream regulators demonstrated concordant predicted inhibition or activation between datasets, the downstream molecules contributing to these predictions only partially overlapped between transcriptomic and proteomic analysis (Supplemental Tables 4, 7, & 8).

## Discussion

Although skin cancer is a well-established outcome of long-term iAs exposure, the role of arsenic metabolism in mediating this process remains unclear. Acute MMA^III^ exposure is more cytotoxic than iAs^III^ in HaCaT cells (Hwang et al. 2024), and epidemiological studies link higher percent MMA in urine or toenail with increased skin cancer risk, suggesting a potential role for methylated arsenic species (Hood et al. 2023; Speer et al. 2023; Yu et al. 2000). However, arsenic speciation has not been directly measured in skin tissue from chronically exposed populations. Consequently, it remains unclear whether human keratinocytes methylate arsenic in vivo and, if so, which arsenic metabolite contributes to skin carcinogenesis.

We demonstrate that arsenic metabolism deficiency is a shared feature across multiple human skin keratinocytes, including primary keratinocytes (HEKn) and the immortalized HaCaT and Ker-CT cell lines (Fig. 1 and Supplemental Fig. 1). The comparable lack of arsenic methylation across primary and immortalized models supports the relevance of HaCaT cells for studying iAs^III^-induced skin carcinogenesis and is consistent with prior reports showing no detectable arsenic metabolites in HaCaT cells and in excised human tissue (Ouypornkochagorn and Feldmann 2010; Pi et al. 2008). Together, these findings indicate that iAs^III^, rather than methylated metabolites, is likely the dominant species driving iAs-induced skin carcinogenesis.

Mechanistically, limited arsenic methylation in keratinocytes is explained by minimal to undetectable AS3MT expression (Fig. 1D). Canonical AS3MT transcripts were extremely low or undetectable across all keratinocyte models, consistent with our prior transcriptomic and proteomic analyses (Banerjee et al. 2021). Interestingly, we identified a novel intron 2-retaining AS3MT isoform containing a premature termination codon that is likely to undergo nonsense-mediated decay without producing a functional protein (Fig. 1D & Supplementary Fig. 2). The absence of corresponding peptide in our proteomic data supports this interpretation. Differences in AS3MT mRNA isoform abundance between HepG2 and HaCaT cells (Fig. 1D) likely result from regulatory unproductive splicing and translation (RUST), providing a mechanism for tissue-specific suppression of arsenic methylation in skin (Zavileyskiy and Pervouchine 2024).

Chronic exposure of HaCaT cells to 100 nM iAs^III^ for 28 weeks produces a well-characterized cSCC-like phenotype and reflects environmentally relevant human plasma concentrations (Pi et al. 2008). Use of 100 nM iAs concentration is critical given the nonlinear, J-shaped dose-response patterns associated with iAs^III^ toxicity (Snow et al. 2005). Accordingly, we examined the proteomic landscape at multiple, critical time points during iAs^III^-induced HaCaT malignant transformation using independently maintained, passage-matched quadruplicate cultures for both unexposed (0 nM iAs^III^) and exposed (100 nM iAs^III^) conditions. Unlike prior single time point studies, this design captures the temporal evolution of protein abundance during malignant progression (Banerjee et al. 2022a, 2024b), while accounting for impacts of passaging time and biological variability. The limited overlap in differentially abundant proteins and pathways across time points highlights that distinct molecular networks operate at different stages of transformation (Figs. 2 and 3; Supplementary Tables 2–3), revealing a highly dynamic proteomic landscape and underscoring the necessity of longitudinal analyses to define mechanisms of cancer initiation and progression (Udensi et al. 2014).

Utilizing this well-established preclinical model and proven quality control measures, robust longitudinal differential protein expression data representing distinct steps in iAs^III^-induced malignant transformation were characterized. Given that hundreds of proteins were differentially abundant at each time point, we sought to integrate the individual protein expression data into biologically meaningful functional relationships relevant to malignant transformation. We achieved this by employing IPA analysis, which extends beyond traditional Gene Ontology enrichment analysis, predicting whether the overrepresented pathways are functionally activated or inhibited (Kramer et al. 2014). IPA analysis provided interesting insights into how iAs^III^ may initiate and promote transformation of keratinocytes.

Genomic instability is both an initiator and driver of carcinogenesis (Ferguson et al. 2015; Requesens et al. 2024). At 7 weeks, Base Excision Repair (BER), and Nucleotide Excision Repair (NER) pathways were predicted to be activated (Supplementary Table 3) consistent with prior reports that low-dose iAs^III^ exposure enhances BER activity (Sykora and Snow 2008). However, pathway activation only reflects increased protein abundance and not necessarily enhanced functional protein activity, as iAs^III^ can displace zinc from critical BER and NER pathway proteins Poly(ADP-Ribose) Polymerase 1 (PARP1) and XPA, DNA damage Recognition and Repair Factor (XPA), respectively, albeit at concentrations ≥ 500 nM (Ding et al. 2017; Vergara-Geronimo et al. 2021). By 19 weeks, BER, NER, and BRCA1-mediated DNA damage signaling were predicted to be inhibited, accompanied by suppression of Ligase 1 (Lig1 expression) (Supplementary Tables 2 & 3). These findings align with previous studies showing that iAs^III^ compromises Lig1 and BRCA1 function, likely promoting reliance on error-prone DNA double-strand break repair (Matthaus et al. 2023; Tam et al. 2020). Collectively, progressive inhibition of DNA repair pathways likely exacerbates genomic instability and contributes to carcinogenic progression and may provide an explanation for mutational signatures within unknown etiology observed in human cancers (Martinez et al. 2013).

Beyond DNA repair, apoptosis signaling was predicted to be inhibited at 19 weeks, consistent with prior studies identifying reduced apoptosis as a driver of iAs^III^-induced malignant transformation in HaCaT cells (Pi et al. 2008; Yang et al. 2024), while metabolic reprogramming (activation of glycolysis and gluconeogenesis) and senescence inhibition emerged at 28 weeks (Supplementary Table 3). These changes reflect classic hallmarks of cancer and suggest a temporal shift from impaired damage signaling and apoptosis towards enhanced survival, proliferation, and metabolic adaptation during iAs-induced malignant transformation (Hanahan 2022).

Notably, two pathways (Spliceosomal Cycle and Cell Cycle Control of Chromosomal Replication) were persistently dysregulated across all time points (Supplementary Table 3), corroborating our prior findings of widespread alternative splicing during iAs^III^-induced transformation (Ferragut Cardoso et al. 2022). The causal role played by aberrant altered splicing in carcinogenesis is well-established (Zhang et al. 2021), reinforcing our observation. Consistent with sustained cell cycle dysregulation, iAs^III^ exposed cells exhibit delayed mitotic progression early during transformation, followed by accelerated proliferation and increased chromosomal instability at later stages (Banerjee et al. 2022b; Lykoudi et al. 2023), Functional dysregulation of splicing, cell cycle and chromosomal instability may be interrelated as iAs^III^ exposure causes significant differential alternative splicing of genes involved in splicing regulation, cell cycle control, and chromosomal organization maintenance (Ferragut Cardoso et al. 2022).

Upstream regulator analysis identified four regulators dysregulated across all time points: CEBPβ, COPS5, KRAS, and E2F1 (Supplementary Table 4). CEBPβ activation is implicated in promoting UVB-induced skin cancer by suppression of Tumor Protein 53 (TP53)-mediated apoptosis in keratinocytes (Tam et al. 2019). Interestingly, predicted activation of CEBPβ function coincided with predicted loss of TP53 function at 7 weeks, aligning with our previous study (Al-Eryani et al. 2018). Although HaCaT cells harbor gain-of-function TP53 mutations (Lehman et al. 1993), they retain partial TP53-associated cell cycle and apoptotic responses (Martynova et al. 2012), suggesting that chronic iAs^III^ exposure may further dysregulate TP53-associated regulatory signaling during malignant transformation. Future studies are required to determine whether loss of CEBPβ can prevent iAs^III^-induced malignant transformation of human skin keratinocytes.

KRAS is one of the most frequently activated oncogenes in human cancer (Huang et al. 2021). Previous work determined that KRAS is overexpressed in arsenic-transformed prostate epithelial cells, with evidence suggesting that this overexpression is driven by retroviral-mediated KRAS gene amplification and expression as opposed to activating KRAS mutations (Merrick et al. 2020). Consistent with these findings, our data predict sustained activation of KRAS function throughout the timeline of malignant transformation, indicating that continuous KRAS activation is an early molecular event necessary for iAs^III^-induced malignant transformation (Supplementary Table 4). Notably, KRAS-activating mutations are not typically associated with UV-induced non-melanoma skin cancers (van der Schroeff et al. 1990), suggesting that enhanced KRAS activation without underlying mutations may be a distinguishing feature of iAs- versus UV-induced non-melanoma skin cancers.

Consistent with minimal arsenic methylation in human keratinocytes, C3H1 and C4-type zinc finger proteins were enriched among differentially abundant proteins (Supplementary Table 5) and dysregulated upstream regulators (Supplementary Table 6). Notably, 87% of differentially abundant zinc finger proteins (66/76) contained C3H1 or C4 motifs, compared to only 10 differentially abundant C2H2 zinc finger proteins. Similarly, 81% of predicted dysregulated zinc finger upstream regulators (26/32) contained C3H1 or C4 motifs. Because iAs^III^ selectively binds and displaces zinc from C3H1/C4 zinc finger motifs (Zhou et al. 2011, 2014, 2021), these findings support a model in which iAs^III^ drives keratinocyte transformation primarily through selective disruption of C3H1/C4 zinc finger regulatory proteins, rather than through methylated arsenic metabolites that broadly target all zinc finger classes (Zhou et al. 2014).

Dysregulated C3H1/C4 zinc finger regulatory proteins provide vital clues towards understanding how iAs^III^ might be transforming keratinocytes. Estrogen Receptor 1 (ESR1; also known as ERα) function was predicted to be activated at 7 weeks (Supplementary Table 6 and Supplementary Fig. 3A), aligning with prior reports of increased ERα activity after iAs^III^ exposure (Stoica et al. 2000). Given that ligand-bound ERα shortens cell cycle progression and induces DNA double-strand breaks during S phase (JavanMoghadam et al. 2016), the impact of iAs^III^-induced ERα activity on cell cycle dynamics during malignant transformation warrants further investigation. PML regulatory function was also predicted to be suppressed at 19- and 28 weeks, consistent with our observation of significantly reduced PML protein expression following chronic iAs exposure (Supplementary Tables 2 & 6and Supplementary Fig. 3B–C). Acute therapeutic concentrations of iAs^III^ (arsenic trioxide; ATO) bind to the PML zinc finger motif of the PML-RARα fusion protein in acute promyelocytic leukemia, leading to its degradation (Ananta et al. 2023). Interestingly, RARα expression was also significantly reduced in malignantly transformed HaCaT cells (Supplementary Table 1). The effects of environmentally relevant iAs^III^ (100 nM) exposure on wild-type PML and RARα protein remain uncharacterized. Given that loss of PML function impairs apoptosis and senescence-associated signaling (Guan and Kao 2015), persistant, predicted suppression of PML function at both 19- and 28-weeks may reflect broader disruption of tumor suppressive stress response networks during iAs^III^-induced malignant transformation. Our findings identify PML as a candidate regulatory factor within dynamically evolving transformation-associated signaling networks rather than as an isolated driver of transformation. Future studies are required to determine whether altered PML-associated signaling functionally contributes to network remodeling during iAs^III^-induced malignant transformation.

Interestingly, both predicted activation and inhibition of C3H1/C4 zinc finger-containing upstream regulators were observed following chronic iAs^III^ exposure (Fig. 5). These findings suggest that iAs^III^ interactions with zinc finger-containing proteins may not uniformly suppress protein regulatory activity. Depending on the affected protein, direct binding and displacement of Zn by iAs^III^ may alter protein conformation, protein-protein interactions, transcriptional activity, protein stability, or subcellular localization in distinct ways. Furthermore, because upstream regulator activity predictions are inferred from downstream molecular expression patterns, disruption of one zinc finger-dependent regulatory pathway may contribute to activation of interconnected regulatory networks during malignant transformation. For example, PHF12 function was predicted to be inhibited after 7 weeks of iAs^III^ exposure. As a component of the SIN3B histone deacetylase complex, reduced PHF12 activity may alter SIN3B-mediated transcriptional repression, resulting in continued expression of genes that may be regulatory targets of other upstream regulators (Wan et al. 2023). Collectively, these findings support a model in which iAs^III^ exposure dynamically reshapes zinc finger-dependent signaling networks rather than uniformly suppressing zinc finger protein function.

Several upstream regulators were predicted to be inhibited or activated by chronic iAs^III^ exposure at the same time point based on transcriptomic and proteomic analyses including PHF12, TP53, NUPR1 (7 weeks; inhibited), E2F1 (7 weeks; activated) and FOXO3, VDR, and CEBPB (19 weeks; inhibited). However, downstream molecules contributing to concordant upstream regulator predictions between transcriptomic and proteomic datasets only partially overlapped. This partial overlap may reflect differences in transcript stability, translational regulation, protein turnover, and analytical depth between transcriptomic and proteomic approaches, but may also reflect broader regulatory functions associated with a given protein. For example, TP53 possess both transcription-dependent and transcription-independent functions dependent on post-translational modifications that regulate protein stability, turnover, cellular localization, and protein-protein interactions (Ho et al. 2019). Collectively, these findings highlight the multilayered complexity of regulatory remodeling during iAs^III^-induced malignant transformation and suggest that similar transformation-associated regulatory states may emerge through distinct transcriptomic and proteomic alterations.

Despite the breadth of the current study, limitations must be addressed. First, our workflow captured steady-state protein abundance at discrete time points, providing stage-specific insight but representing only snapshots of a dynamic transformation process (Banerjee et al. 2021). It is also important to note that population-averaged measurements may not reflect changes occurring specifically within transformed or initiated cells, which are likely a small minority of cells within the mixed cell population exposed to iAs^III^. Second, proteomics preferentially detects moderately to highly abundant proteins, potentially underrepresenting low-abundance regulators relevant for carcinogenesis (Shi et al. 2012). Third, we applied lenient criteria to identify differential expression (*p* < 0.05; no fold-change threshold) to maximize pathway coverage and biological interpretability, consistent with prior studies (Ekuban et al. 2025). Notably, this approach yielded robust predictions, with 59 canonical pathways and 344 upstream regulators exceeding Z-score > I1I activation threshold, reflecting coordinated expression changes among pathway members (Supplementary Tables 3 & 4) (Kramer et al. 2014). Fourth, our workflow does not capture post-translational modifications which can critically influence protein function. For example, TP53 was not differentially expressed (Supplementary Table 2) but was predicted to be functionally inhibited after 7 weeks iAs^III^ exposure based on both transcriptomic and proteomic data (Supplementary Tables 4 & 7), consistent with our prior work showing iAs^III^-dependent suppression of TP53 activity via altered phosphorylation and acetylation (Al-Eryani et al. 2018). Future work incorporating increased temporal resolution, post-translationally focused proteomics, and integrative multi-omic analyses will be essential to precisely define the required sequence of molecular events driving iAs-induced carcinogenesis.

**Fig. 6:**
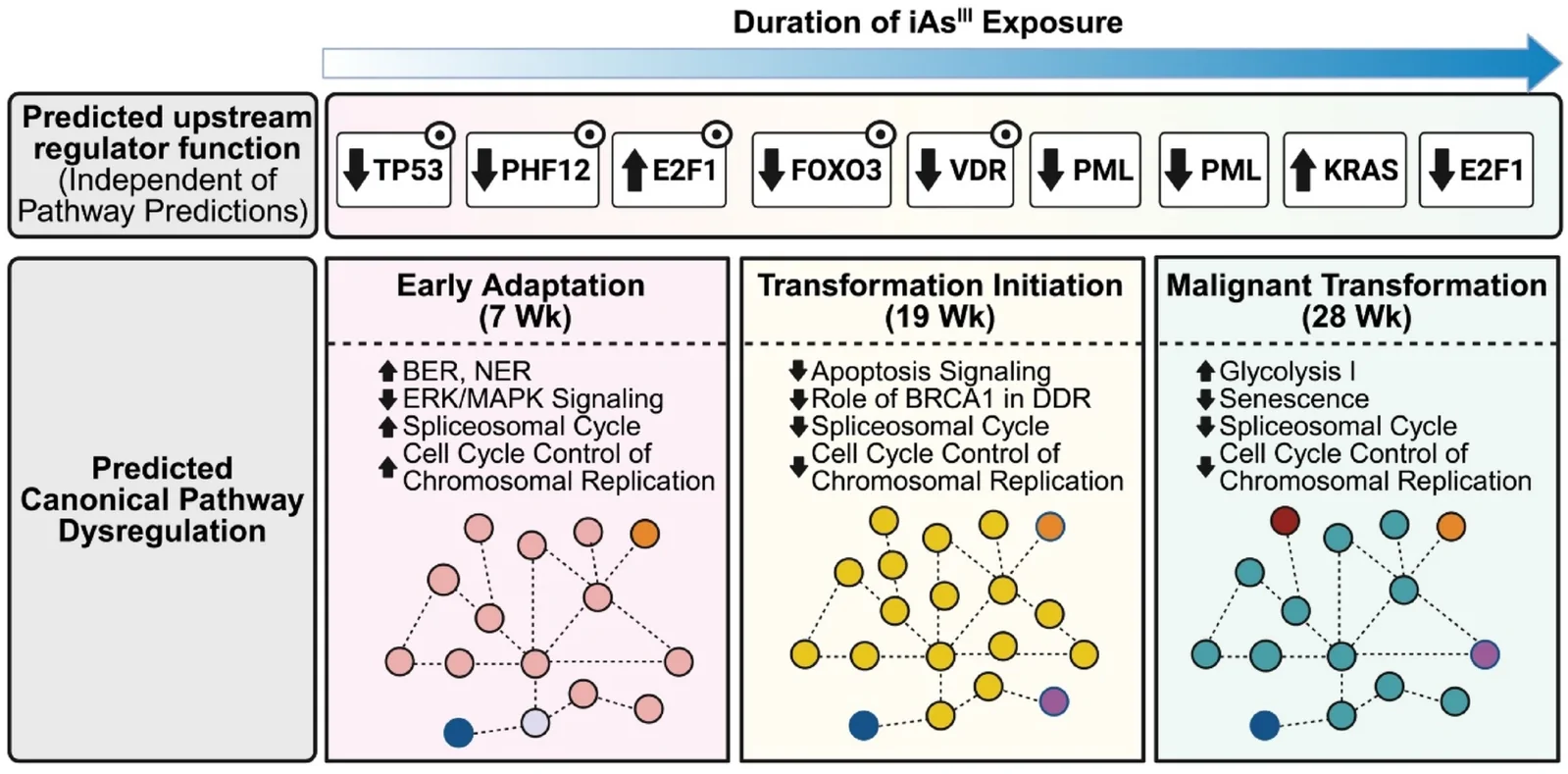
Dynamic remodeling of predicted regulatory and canonical pathway programs during iAs^III^-induced malignant transformation. Predicted upstream regulator function and canonical pathway dysregulation were evaluated at distinct stages during iAs^III^-induced malignant transformation of human keratinocytes by IPA. Examples of predicted dysregulation and inferred regulator activity are shown independently across early adaptation (7 wk), transformation initiation (19 wk), and malignant transformation (28 wk). Regulators marked with a circle were concordantly predicted by both transcriptomic and proteomic analyses at the same time point. Colored pathway nodes represent pathways/programs rather than individual proteins and are included to illustrate dynamic remodeling and persistence of altered biological processes across transformation stages. Note that most predicted pathways dysregulated are unique to each time point, but some pathway dysregulation is shared across timepoints and is illustrated as similar colored circles (orange, dark blue, purple). Upward arrows indicate predicted activation whereas arrows pointing down indicate inhibition of the indicated pathway or regulator. This model summarizes inferred relationship from integrated omics analyses and pathway modeling and does not imply direct mechanistic causality. At 7 wks, increased Base Excision Repair (BER) and Nucleotide Excision Repair (NER) combined with decreased ERK/MAPK signaling may reflect selection for iAs^III^ resistant cell populations. At 19 wks, decreased apoptosis signaling combined with reduced BRCA1-mediated DNA Damage Response (DDR) signaling suggests progressive loss of genome maintenance and survival checkpoint regulation during transformation initiation. At 28 wks, increased glycolytic signaling and suppression of senescence-associated pathways are consistent with establishment of a transformed phenotype. Created in BioRender

In summary, this study defines how chronic iAs^III^ exposure longitudinally reshapes the keratinocyte proteome in a well-established preclinical model of iAs-induced cSCC, while clarifying the contribution of limited arsenic metabolism to these changes. Our data reveal a coordinated, dynamic global rewiring of pathways during iAs^III^-induced malignant transformation. By integrating arsenic metabolism, proteome remodeling, and predicted upstream regulator activity, we provide further insight into how chronic iAs^III^ exposure promotes keratinocyte malignant transformation. This work establishes a robust model of stepwise transformation initiation and progression in human keratinocytes (Fig. 6) and identifies a focused set of C3H1- and C4-type zinc finger proteins as likely mediators of iAs^III^-induced transformation (Supplementary Tables 5 & 6). Furthermore, our data indicating functional inactivation of tumor suppressors demonstrate how an environmental exposure can potentially mimic tumor suppressor loss-of-function mutations as a mechanism contributing to iAs^III^-induced malignant transformation.

## Supplementary Information

Below is the link to the electronic supplementary material.

Supplementary Material 1

Supplementary Material 2

Supplementary Material 3

Supplementary Material 4

Supplementary Material 5

Supplementary Material 6

Supplementary Material 7

Supplementary Material 8

Supplementary Material 9

## Data Availability

All proteomic and sequencing data have been deposited to publicly available databases (accession numbers are provided in the materials and methods sections). All other data/material generated as a part of this work are available upon request.

## References

[CR1] Al-Eryani L, Waigel S, Tyagi A et al (2018) Differentially expressed mRNA targets of differentially expressed miRNAs predict changes in the TP53 axis and carcinogenesis-related pathways in human keratinocytes chronically exposed to arsenic. Toxicol Sci 162(2):645–654. 10.1093/toxsci/kfx292 PMC5889014 29319823

[CR2] Alexander ET, Mariner K, Borodyanskaya Y, Minton A, Gilmour SK (2019) Polyamine-stimulation of arsenic-transformed keratinocytes. Carcinogenesis 40(8):1042–1051. 10.1093/carcin/bgz115 PMC6735862 31190067

[CR3] Ananta, Benerjee S, Tchounwou PB, Kumar S (2023) Mechanistic update of trisenox in blood cancer. Curr Res Pharmacol Drug Discov 5:100166. 10.1016/j.crphar.2023.100166 PMC10701371 38074774

[CR4] Bajsert J, De Glas V, Faway E et al (2024) Characterization of reconstructed human epidermis in a chemically-defined, animal origin-free cell culture. JID Innov 4(5):100298. 10.1016/j.xjidi.2024.100298 PMC11327582 39157429

[CR6] Banerjee M, Ferragut Cardoso A, Al-Eryani L et al (2021) Dynamic alteration in miRNA and mRNA expression profiles at different stages of chronic arsenic exposure-induced carcinogenesis in a human cell culture model of skin cancer. Arch Toxicol 95(7):2351–2365. 10.1007/s00204-021-03084-2 PMC8241660 34032870

[CR5] Banerjee M, Al-Eryani L, Srivastava S et al (2022a) Delineating the effects of passaging and exposure in a longitudinal study of arsenic-induced squamous cell carcinoma in a HaCaT cell line model. Toxicol Sci 185(2):184–196. 10.1093/toxsci/kfab129 PMC8795903 34730829

[CR9] Banerjee M, Yaddanapudi K, States JC (2022b) Zinc supplementation prevents mitotic accumulation in human keratinocyte cell lines upon environmentally relevant arsenic exposure. Toxicol Appl Pharmacol 454:116255. 10.1016/j.taap.2022.116255 PMC9683715 36162444

[CR7] Banerjee M, Lykoudi A, Hwang JY et al (2024a) Dysregulation of mRNA expression by hsa-miR-186 overexpression in arsenic-induced skin carcinogenesis. Toxicol Appl Pharmacol 495:117209. 10.1016/j.taap.2024.117209 PMC12052204 39719251

[CR8] Banerjee M, Srivastava S, Rai SN, States JC (2024b) Chronic arsenic exposure induces malignant transformation of human HaCaT cells through both deterministic and stochastic changes in transcriptome expression. Toxicol Appl Pharmacol 484:116865. 10.1016/j.taap.2024.116865 PMC10994602 38373578

[CR10] Barajas-Olmos FM, Ortiz-Sanchez E, Imaz-Rosshandler I et al (2019) Analysis of the dynamic aberrant landscape of DNA methylation and gene expression during arsenic-induced cell transformation. Gene 711:143941. 10.1016/j.gene.2019.143941 31242453

[CR11] Bercier P, Wang QQ, Zang N et al (2023) Structural basis of PML-RARA oncoprotein targeting by arsenic unravels a cysteine rheostat controlling pml body assembly and function. Cancer Discov 13(12):2548–2565. 10.1158/2159-8290.CD-23-0453 PMC10714139 37655965

[CR12] Boukamp P, Petrussevska RT, Breitkreutz D, Hornung J, Markham A, Fusenig NE (1988) Normal keratinization in a spontaneously immortalized aneuploid human keratinocyte cell line. J Cell Biol 106(3):761–771. 10.1083/jcb.106.3.761 PMC2115116 2450098

[CR13] Calatayud M, Devesa V, Velez D (2013) Differential toxicity and gene expression in Caco-2 cells exposed to arsenic species. Toxicol Lett 218(1):70–80. 10.1016/j.toxlet.2013.01.013 23353816

[CR14] Ding X, Zhou X, Cooper KL, Huestis J, Hudson LG, Liu KJ (2017) Differential sensitivities of cellular XPA and PARP-1 to arsenite inhibition and zinc rescue. Toxicol Appl Pharmacol 331:108–115. 10.1016/j.taap.2017.05.031 PMC5568456 28552776

[CR15] Drobna Z, Xing W, Thomas DJ, Styblo M (2006) shRNA silencing of AS3MT expression minimizes arsenic methylation capacity of HepG2 cells. Chem Res Toxicol 19(7):894–898. 10.1021/tx060076u PMC2329798 16841956

[CR16] Ekuban FA, Gripshover TC, Ames P et al (2025) Perfluorooctane sulfonate exposure and alcohol-associated liver disease severity in a mouse chronic-binge ethanol feeding model. Toxicol Sci 206(2):388–408. 10.1093/toxsci/kfaf066 PMC12329793 40347464

[CR17] Ferguson LR, Chen H, Collins AR et al (2015) Genomic instability in human cancer: Molecular insights and opportunities for therapeutic attack and prevention through diet and nutrition. Semin Cancer Biol 35(SupplSuppl):S5–S24. 10.1016/j.semcancer.2015.03.005 PMC4600419 25869442

[CR18] Ferragut Cardoso AP, Banerjee M, Al-Eryani L et al (2022) Temporal modulation of differential alternative splicing in HaCaT human keratinocyte cell line chronically exposed to arsenic for up to 28 Wk. Environ Health Perspect 130(1):17011. 10.1289/EHP9676 PMC8785870 35072517

[CR19] Gasteiger E, Gattiker A, Hoogland C, Ivanyi I, Appel RD, Bairoch A (2003) ExPASy: the proteomics server for in-depth protein knowledge and analysis. Nucleic Acids Res 31(13):3784–3788. 10.1093/nar/gkg563 PMC168970 12824418

[CR20] Guan D, Kao HY (2015) The function, regulation and therapeutic implications of the tumor suppressor protein, PML. Cell Biosci 5:60. 10.1186/s13578-015-0051-9 PMC4632682 26539288

[CR21] Guo Z, Hu Q, Tian J et al (2016) Proteomic profiling reveals candidate markers for arsenic-induced skin keratosis. Environ Pollut 218:34–38. 10.1016/j.envpol.2016.08.035 27552035

[CR22] Hanahan D (2022) Hallmarks of cancer: new dimensions. Cancer Discov 12(1):31–46. 10.1158/2159-8290.CD-21-1059 35022204

[CR23] HGNC Gene Group Zinc Fingers. In: Available via European Molecular Biology Laboratory, European Bioinformatics Institute (EMBL-EBI). https://www.genenames.org/data/genegroup/#!/group/26. Accessed 02 Feb 2023

[CR24] Ho T, Tan BX, Lane D (2019) How the other half lives: what p53 Does when it is not being a transcription factor. Int J Mol Sci. 10.3390/ijms21010013 PMC6982169 31861395

[CR25] Hood KM, Sweeney E, Ilie G, Keltie E, Kim JS (2023) Toenail arsenic species and metallome profiles associated with breast, cervical, prostate, and skin cancer prevalence in the Atlantic Partnership for Tomorrow’s Health cohort. Front Public Health 11:1148283. 10.3389/fpubh.2023.1148283 PMC10308375 37397723

[CR26] Huang L, Guo Z, Wang F, Fu L (2021) KRAS mutation: from undruggable to druggable in cancer. Signal Transduct Target Ther 6(1):386. 10.1038/s41392-021-00780-4 PMC8591115 34776511

[CR27] Hwang JH, An GJ, Kim CH, Chung HY, Lim KM (2024) Trivalent arsenicals induce skin toxicity through thiol depletion. Toxicol Appl Pharmacol 492:117115. 10.1016/j.taap.2024.117115 39357682

[CR28] JavanMoghadam S, Weihua Z, Hunt KK, Keyomarsi K (2016) Estrogen receptor alpha is cell cycle-regulated and regulates the cell cycle in a ligand-dependent fashion. Cell Cycle 15(12):1579–1590. 10.1080/15384101.2016.1166327 PMC4934046 27049344

[CR29] Kervestin S, Jacobson A (2012) NMD: a multifaceted response to premature translational termination. Nat Rev Mol Cell Biol 13(11):700–712. 10.1038/nrm3454 PMC3970730 23072888

[CR30] Kramer A, Green J, Pollard J Jr., Tugendreich S (2014) Causal analysis approaches in ingenuity pathway analysis. Bioinformatics 30(4):523–530. 10.1093/bioinformatics/btt703 PMC3928520 24336805

[CR31] Lehman TA, Modali R, Boukamp P et al (1993) p53 mutations in human immortalized epithelial cell lines. Carcinogenesis 14(5):833–839. 10.1093/carcin/14.5.833 8504475

[CR32] Lykoudi A, Ferragut Cardoso AP, Wise SS, Banerjee M, States JC (2023) Clonal variability in chromosomal instability as a potential driver in the acquisition of tumorigenic phenotype in chronic arsenic-exposed and hsa-miR-186 overexpressing human keratinocytes. Toxicol Appl Pharmacol 479:116730. 10.1016/j.taap.2023.116730 PMC10866375 37866707

[CR33] Martinez VD, Thu KL, Vucic EA et al (2013) Whole-genome sequencing analysis identifies a distinctive mutational spectrum in an arsenic-related lung tumor. J Thorac Oncol 8(11):1451–1455. 10.1097/JTO.0b013e3182a4dd8e 24128716

[CR34] Martynova E, Pozzi S, Basile V et al (2012) Gain-of-function p53 mutants have widespread genomic locations partially overlapping with p63. Oncotarget 3(2):132–143. 10.18632/oncotarget.447 PMC3326644 22361592

[CR35] Matousek T, Wang Z, Douillet C, Musil S, Styblo M (2017) Direct speciation analysis of arsenic in whole blood and blood plasma at low exposure levels by hydride generation-cryotrapping-inductively coupled plasma mass spectrometry. Anal Chem 89(18):9633–9637. 10.1021/acs.analchem.7b01868 PMC6611167 28809551

[CR36] Matthaus T, Stosser S, Seren HY, Haberland VMM, Hartwig A (2023) Arsenite impairs BRCA1-dependent DNA double-strand break repair, a mechanism potentially contributing to genomic instability. Int J Mol Sci. 10.3390/ijms241814395 PMC10532266 37762697

[CR37] Medina S, Bolt AM, Zhou X et al (2021) Arsenite and monomethylarsonous acid disrupt erythropoiesis through combined effects on differentiation and survival pathways in early erythroid progenitors. Toxicol Lett 350:111–120. 10.1016/j.toxlet.2021.07.008 PMC8487637 34274428

[CR38] Merrick BA, Phadke DP, Bostrom MA et al (2020) KRAS-retroviral fusion transcripts and gene amplification in arsenic-transformed, human prostate CAsE-PE cancer cells. Toxicol Appl Pharmacol 397:115017. 10.1016/j.taap.2020.115017 PMC7606314 32344290

[CR39] Mir SA, Pinto SM, Paul S et al (2017) SILAC-based quantitative proteomic analysis reveals widespread molecular alterations in human skin keratinocytes upon chronic arsenic exposure. Proteomics. 10.1002/pmic.201600257 28000977

[CR40] Nail AN, McCaffrey LM, Banerjee M, Ferragut Cardoso AP, States JC (2022) Chronic arsenic exposure suppresses ATM pathway activation in human keratinocytes. Toxicol Appl Pharmacol 446:116042. 10.1016/j.taap.2022.116042 PMC9318262 35513056

[CR41] Ouypornkochagorn S, Feldmann J (2010) Dermal uptake of arsenic through human skin depends strongly on its speciation. Environ Sci Technol 44(10):3972–3978. 10.1021/es903667y 20415415

[CR42] Pi J, Diwan BA, Sun Y et al (2008) Arsenic-induced malignant transformation of human keratinocytes: involvement of Nrf2. Free Radic Biol Med 45(5):651–658. 10.1016/j.freeradbiomed.2008.05.020 PMC2658531 18572023

[CR43] Reaves ML, Sinha S, Rabinowitz JD, Kruglyak L, Redfield RJ (2012) Absence of detectable arsenate in DNA from arsenate-grown GFAJ-1 cells. Science 337(6093):470–473. 10.1126/science.1219861 PMC3845625 22773140

[CR44] Requesens M, Foijer F, Nijman HW, de Bruyn M (2024) Genomic instability as a driver and suppressor of anti-tumor immunity. Front Immunol 15:1462496. 10.3389/fimmu.2024.1462496 PMC11562473 39544936

[CR45] Shah P, Trinh E, Qiang L et al (2017) Arsenic induces p62 expression to form a positive feedback loop with Nrf2 in human epidermal keratinocytes: implications for preventing arsenic-induced skin cancer. Molecules. 10.3390/molecules22020194 PMC5361890 28125038

[CR46] Shi T, Su D, Liu T et al (2012) Advancing the sensitivity of selected reaction monitoring-based targeted quantitative proteomics. Proteomics 12(8):1074–1092. 10.1002/pmic.201100436 PMC3375056 22577010

[CR47] Smeester L, Bommarito PA, Martin EM et al (2017) Chronic early childhood exposure to arsenic is associated with a TNF-mediated proteomic signaling response. Environ Toxicol Pharmacol 52:183–187. 10.1016/j.etap.2017.04.007 PMC5796657 28433805

[CR48] Snow ET, Sykora P, Durham TR, Klein CB (2005) Arsenic, mode of action at biologically plausible low doses: what are the implications for low dose cancer risk? Toxicol Appl Pharmacol 207(2 Suppl):557–564. 10.1016/j.taap.2005.01.048 15996700

[CR49] Speer RM, Zhou X, Volk LB, Liu KJ, Hudson LG (2023) Arsenic and cancer: evidence and mechanisms. Adv Pharmacol 96:151–202. 10.1016/bs.apha.2022.08.001 PMC10860672 36858772

[CR50] Spivak G, Hanawalt PC (1995) Determination of damage and repair in specific DNA sequences. Methods 7(2):147–161. 10.1006/meth.1995.1021

[CR51] Stoica A, Pentecost E, Martin MB (2000) Effects of arsenite on estrogen receptor-alpha expression and activity in MCF-7 breast cancer cells. Endocrinology 141(10):3595–3602. 10.1210/endo.141.10.7704 11014213

[CR52] Su PF, Hu YJ, Ho IC, Cheng YM, Lee TC (2006) Distinct gene expression profiles in immortalized human urothelial cells exposed to inorganic arsenite and its methylated trivalent metabolites. Environ Health Perspect 114(3):394–403. 10.1289/ehp.8174 PMC1392234 16507463

[CR53] Sun Y, Pi J, Wang X, Tokar EJ, Liu J, Waalkes MP (2009) Aberrant cytokeratin expression during arsenic-induced acquired malignant phenotype in human HaCaT keratinocytes consistent with epidermal carcinogenesis. Toxicology 262(2):162–170. 10.1016/j.tox.2009.06.003 PMC2747077 19524636

[CR54] Sykora P, Snow ET (2008) Modulation of DNA polymerase beta-dependent base excision repair in cultured human cells after low dose exposure to arsenite. Toxicol Appl Pharmacol 228(3):385–394. 10.1016/j.taap.2007.12.019 18252256

[CR55] Tam HW, Hall JR, Messenger ZJ et al (2019) C/EBPbeta suppresses keratinocyte autonomous type 1 IFN response and p53 to increase cell survival and susceptibility to UVB-induced skin cancer. Carcinogenesis 40(9):1099–1109. 10.1093/carcin/bgz012 PMC10893916 30698678

[CR56] Tam LM, Price NE, Wang Y (2020) Molecular mechanisms of arsenic-induced disruption of DNA repair. Chem Res Toxicol 33(3):709–726. 10.1021/acs.chemrestox.9b00464 PMC7078036 31986875

[CR57] Tokar EJ, Benbrahim-Tallaa L, Ward JM, Lunn R, Sams RL, Waalkes MP (2010) Cancer in experimental animals exposed to arsenic and arsenic compounds. Crit Rev Toxicol 40(10):912–927. 10.3109/10408444.2010.506641 PMC3076186 20812815

[CR58] Udensi UK, Tackett AJ, Byrum S et al (2014) Proteomics-based identification of differentially abundant proteins from human keratinocytes exposed to arsenic trioxide. J Proteom Bioinform 7(7):166–178. 10.4172/jpb.1000317 PMC4240501 25419056

[CR59] van der Schroeff JG, Evers LM, Boot AJ, Bos JL (1990) Ras oncogene mutations in basal cell carcinomas and squamous cell carcinomas of human skin. J Invest Dermatol 94(4):423–425. 10.1111/1523-1747.ep12874504 2179417

[CR60] Vergara-Geronimo CA, Del Leon A, Rodriguez-Dorantes M, Ostrosky-Wegman P, Salazar AM (2021) Arsenic-protein interactions as a mechanism of arsenic toxicity. Toxicol Appl Pharmacol 431:115738. 10.1016/j.taap.2021.115738 34619159

[CR61] Wan MSM, Muhammad R, Koliopoulos MG, Roumeliotis TI, Choudhary JS, Alfieri C (2023) Mechanism of assembly, activation and lysine selection by the SIN3B histone deacetylase complex. Nat Commun 14(1):2556. 10.1038/s41467-023-38276-0 PMC10156912 37137925

[CR62] Yang Q, Zhang T, Liu J et al (2024) Sustained high expression of NRF2 inhibits cell apoptosis in arsenite-transformed human keratinocytes. Food Chem Toxicol 191:114875. 10.1016/j.fct.2024.114875 39033872

[CR63] Yu RC, Hsu KH, Chen CJ, Froines JR (2000) Arsenic methylation capacity and skin cancer. Cancer Epidemiol Biomarkers Prev 9(11):1259–1262 11097236

[CR64] Zavileyskiy LG, Pervouchine DD (2024) Post-transcriptional regulation of gene expression via unproductive splicing. Acta Naturae 16(1):4–13. 10.32607/actanaturae.27337 PMC11062102 38698955

[CR65] Zhang F, Paramasivam M, Cai Q et al (2014) Arsenite binds to the RING finger domains of RNF20-RNF40 histone E3 ubiquitin ligase and inhibits DNA double-strand break repair. J Am Chem Soc 136(37):12884–12887. 10.1021/ja507863d PMC4183597 25170678

[CR66] Zhang Y, Qian J, Gu C, Yang Y (2021) Alternative splicing and cancer: a systematic review. Signal Transduct Target Ther 6(1):78. 10.1038/s41392-021-00486-7 PMC7902610 33623018

[CR68] Zhou X, Sun X, Cooper KL, Wang F, Liu KJ, Hudson LG (2011) Arsenite interacts selectively with zinc finger proteins containing C3H1 or C4 motifs. J Biol Chem 286(26):22855–22863. 10.1074/jbc.M111.232926 PMC3123053 21550982

[CR69] Zhou X, Sun X, Mobarak C et al (2014) Differential binding of monomethylarsonous acid compared to arsenite and arsenic trioxide with zinc finger peptides and proteins. Chem Res Toxicol 27(4):690–698. 10.1021/tx500022j PMC3998772 24611629

[CR67] Zhou X, Speer RM, Volk L, Hudson LG, Liu KJ (2021) Arsenic co-carcinogenesis: inhibition of DNA repair and interaction with zinc finger proteins. Semin Cancer Biol 76:86–98. 10.1016/j.semcancer.2021.05.009 PMC8578584 33984503

