# Supplementary material for: Deficient arsenic methylation and global proteomic reprogramming in human keratinocytes during arsenic-induced skin carcinogenesis
Source: Arch Toxicol. 2026 Jun 27;100(11):5053–70. doi: 10.1007/s00204-026-04478-w (PMC13546112; doi:10.1007/s00204-026-04478-w)
Supplement: Supplementary file 1 [file 204_2026_4478_MOESM9_ESM.docx]

Supplementary Figure 1.

**Supplementary Figure 1. Methylated metabolites are near-absent from human keratinocyte cultures, including both culture media and cells.** Percent methylated arsenic relative to total arsenic detected in culture plates following acute iAs^III^ exposure (24 h, 100 nM) was quantified in HepG2 hepatocellular carcinoma and human keratinocyte cell models (HEKn, Ker-CT, and HaCaT). HepG2 cultures demonstrated substantially higher arsenic methylation capacity compared to all keratinocyte models, whereas methylated arsenic species were near-absent in HEKn, Ker-CT, and HaCaT cultures. Data are presented as mean ± SD from technical replicates. Statistical significance was determined by one-way ANOVA followed by Tukey’s multiple comparisons test. **** p < 0.0001.

Supplementary Figure 2.

**Supplementary Figure 2. Detection of a premature stop codon containing novel intron retention AS3MT mRNA isoform.** **A.** Primers (forward primer and reverse primer) were designed to exclusively amplify canonical AS3MT mRNA (AS3MT-201) while excluding all other annotated AS3MT isoforms including AS3MT d2d3 (exon 2 and exon 3 skipping AS3MT splice transcript) and BORCS7-AS3MT (read-through transcript from the upstream *BORCS7* gene). Specifically, the forward primer spanned Exon 1 and 2 junction of the AS3MT canonical mRNA, which is absent from the BORCS7-AS3MT transcript, whereas the reverse primer was in exon 3 of the canonical AS3MT transcript, which is absent from the AS3MT d2d3 transcript. Cloning and sequencing of products from RT-PCR led to the discovery of a novel intron 2 (i2; orange line) retaining AS3MT mRNA transcript (i2-AS3MT; Figure 1D; 332 bp amplicon). **B.** Alignment of canonical AS3MT (AS3MT-201) and intron 2 retaining novel AS3MT mRNA (i2-AS3MT). Virtual translation using Expasy tool shows that intron 2 retention results in a predicted reading frame that is out of frame with AS3MT exons 3 and 4 and contains a premature stop codon in exon 4 (indicated by * and stop sign). Thus, this intron retaining isoform is likely degraded by nonsense mediated decay.

Supplementary Figure 3.

**Supplementary Figure 3. Chronic iAs^III^ exposure dysregulates the regulatory function of its established targets during malignant transformation. A.** ESR1 function is predicted to be activated (orange fill) following chronic iAs exposure (100 nM) at 7 weeks. **B - C.** PML function is predicted to be inhibited (blue fill) following chronic iAs exposure (100 nM) at 19 and 28 weeks. For all maps, the upstream regulator is in the center of the regulation map and colored based on the confidence of the prediction for functional activation (orange) or functional inhibition (blue). Established downstream targets for each upstream regulator (ESR1/PML) that are differentially abundant in our dataset at each time point are shown. Color gradient corresponds to log_2_(Fold Change) abundant values for each protein (red upregulated; green downregulated). Also shown are the predicted functional relationships between the dysregulated upstream regulator and downstream differentially abundant target proteins (activation: orange arrow; inhibition: blue T bars; inconsistent with published literature: yellow arrows or T bars; or not predicted: gray arrows, either solid or dotted lined).
